# Prognostic significance of E-cadherin and N-cadherin expression in Gliomas

**DOI:** 10.1186/s12885-017-3591-z

**Published:** 2017-08-29

**Authors:** Myung-Giun Noh, Se-Jeong Oh, Eun-Jung Ahn, Yeong-Jin Kim, Tae-Young Jung, Shin Jung, Kyung-Keun Kim, Jae-Hyuk Lee, Kyung-Hwa Lee, Kyung-Sub Moon

**Affiliations:** 1 Department of Pathology, Chonnam National University Hwasun Hospital and Medical School, 322 Seoyang-ro, Hwasun-eup, Hwasun-gun, Jeollanam-do 519-763 South Korea; 2 Department of Neurosurgery, Chonnam National University Hwasun Hospital and Medical School, 322 Seoyang-ro, Hwasun-eup, Hwasun-gun, Jeollanam-do 519-763 South Korea; 3 Medical Research Center of Gene Regulation and Center for Creative Biomedical Scientists, Chonnam National University Medical School, Gwangju, South Korea

**Keywords:** E-cadherin, Epithelial-mesenchymal transition, Glioma, N-cadherin, Prognosis, Survival

## Abstract

**Background:**

Epithelial-mesenchymal transition (EMT), principally involving an E-cadherin to N-cadherin shift, linked to tumor invasion or metastasis, and therapeutic resistance in various human cancer. A growing body of recent evidence has supported the hypothesis that EMT play a crucial role in the invasive phenotype of gliomas. To evaluate the prognostic connotation of EMT traits in glioma, expression of E-cadherin and N-cadherin was explored in a large series of glioma patients in relation to patient survival rate.

**Methods:**

Expressions of E- and N-cadherin were examined using immunohistochemical analysis in 92 glioma cases diagnosed at our hospital. These markers expressions were also explored in 21 cases of fresh frozen glioma samples and in glioma cell lines by Western blot analysis.

**Results:**

Expression of E-cadherin was observed in eight cases (8.7%) with weak staining intensity in the majority of the immunoreactive cases (7/8). Expression of N-cadherin was identified in 81 cases (88.0%) with high expression in 64 cases (69.5%). Fresh frozen tissue samples and glioma cell lines showed similar results by Western blot analysis. There was no significant difference in either overall survival (OS) or progression-free survival (PFS) according to E-cadherin expression (*P* > 0.05). Although the OS rates were not affected by N-cadherin expression levels (*P* = 0.138), PFS increased in the low N-cadherin expression group with marginal significance (*P* = 0.058). The survival gains based on N-cadherin expression levels were significantly augmented in a larger series of publicly available REMBRANDT data (*P* < 0.001).

**Conclusions:**

E- and N-cadherin, as representative EMT markers, have limited prognostic value in glioma. Nonetheless, the EMT process in gliomas may be compounded by enhanced N-cadherin expression supported by unfavorable prognostic outcomes.

## Background

Infiltrative tendency is such a prominent feature of malignant gliomas that tumor cells migrate far from the tumoral epicenter through the surrounding parenchyma [1, 2]. In addition to cytological atypia and mitotic activity that are needed for histopathological definition of lower-grade gliomas, microvascular proliferation and/or necrosis are specific defining attributes of glioblastoma, which is the most malignant primary brain tumor [3]. Even lower-grade gliomas assorted as World Health Organization (WHO) grade II to III are characterized by propensity for diffuse infiltration and for the malignant transformation to higher-grade tumors. The hurdle in therapeutic resistance of gliomas is intimately connected to the infiltrative phenotype. The infiltrative feature is an essential part of the clinical aggravation of malignant glioma, making surgical resection incomplete and promoting regrowth of residual tumor cells [1–3]. Infiltrative phenotypes in epithelial malignancies have been unequivocally linked to the phenomenon of epithelial-mesenchymal transition (EMT) that manifests as tumor recurrence, metastasis and therapeutic intractability [4]. By contrast, only recently has the EMT process in non-epithelial tumors been highlighted as an important player in tumor progression [5–7]. Considering that the various downstream pathways of EMT are related with cancer invasion or metastasis, and therapeutic resistance in non-epithelial human cancer, EMT/EMT-like process can be addressed as a possible therapeutic target [8].

EMT, as a complicated cellular machinery provoked by various circumstantial factors, leads cellular and biochemical acquisition of motile mesenchymal properties from immobile epithelial cells [9]. In recent glioma research, EMT have been named as a key player of tumor progression and invasion. In this respect, the recently defined mesenchymal subgroup of glioblastomas reinforce the idea that the EMT-like process has prognostic consequence for malignant brain tumors [10, 11]. In line with the regulation of stem cell features, EMT may contribute to tumor progression and chemoresistance, and in tumor relapse after treatment as well [8].

Although the contribution of EMT in glioma progression is not as clear as that in epithelial malignancies, glial-mesenchymal transition as a counterpart of EMT has been revealed as an essential process in glioma invasion [12–16]. EMT principally involves an E-cadherin to N-cadherin shift. Still, the clinical impact of E- and N-cadherin including their effect on patient survival rates remains unknown. It is conceivable that expression of E-cadherin and N-cadherin affects the clinical aspect of glioma patients in terms of survival rates. This study assessed the expression of the classic EMT markers in human glioma samples including formalin-fixed paraffin embedded tissues, fresh frozen glioma samples and human and mouse glioma cell lines, and analyzed the implication in the context of patient survival by comparing the data with the statistics from the large cohort NCI Repository for Molecular Brain Neoplasia Database (REMBRANDT).

## Methods

### Glioma cell lines and human glioma tissue specimens

U118, U87, T98G, U343, and U251 human glioma cells lines were from ATCC (Manassas, VA, USA). The GL261 mouse glioma cell line was a gift from Dr. Maciej S. Lesniak at University of Chicago. All cells were maintained and cultured as described previously [12].

Ninety two glioma specimens were obtained from the patient who underwent surgical resection at Chonnam National University Hwasun Hospital between 2007 and 2012. WHO classification of the central nervous system was used for diagnostic criteria [3]. Frozen samples [*N* = 20; 8 low-grade (WHO grades I/II) and 12 high-grade (WHO grades III/IV)] were handled as previously described [12]. Samples obtained within 30 min after surgical resection were frozen immediately using liquid nitrogen. The specimens were stored at −80 °C until used. Clinicopathological data were based on the medical records. Radiological findings, such as size and location of tumor, peritumoral edema, and cystic or necrotic changes were obtained from preoperative magnetic resonance imaging (MRI). Overall survival (OS) and progression-free survival (PFS) were determined as previously described [12]. The endpoint of OS was the date of death/the last follow-up visit. The endpoint of PFS was the date of recurrence/progression/death. The Chonnam National University Hwasun Hospital Institutional Review Board approved this study (CNUHH-2016-081), and written informed consent was obtained from patients or their legal surrogates for using resected glioma samples.

### Tissue microarray construction and immunohistochemistry

Areas with a high cellularity were selected for tissue microarrays. Immunohistochemical staining was performed as described previously [12]. E-cadherin (1:50 dilution; DAKO, Glostrup, Denmark; Catalogue No. M3612) and N-cadherin (1:500 dilution; Abcam, Cambridge, UK; Catalogue No. ab12221) antibodies were applied into a Bond-max autostainer system (Leica Microsystems, Bannockburn, IL, USA). Antigen retrieval was carried out using citrate buffer at pH 6.0. Negative controls were prepared without using primary antibodies.

All immunostained slides were evaluated twice by two independent observers (NMG and LKH) with no knowledge of the clinical details. E- and N-cadherin immunohistochemistry showed cytoplastmic positivity in glioma cells and sometimes stained the cytoplasmic borders. The intensity of staining was initially classified into 4 grades: 0, no immunoreaction; 1, weak positivity; 2, moderate positivity; and 3, strong reactivity. With N-cadherin staining, cases of grades 0 and 1 positivity were grouped as a low-expression, and cases of grades 2 and 3 as a high-expression for statistical convenience. Two pathologists re-evaluated cases with discordant staining intensity together and made concessions for such cases.

### Western blot analysis

Western blot analysis was performed as described [12]. After extraction and quantification of protein from glioma cells and tissues, proteins (30 μg) were resolved by 10% polyacrylamide gel electrophoresis. The proteins were electrotransferred onto nitrocellulose membranes, blocked by 3% skimmed milk, and followed by sequential incubation with primary antibodies (E-cadherin, 1:500 dilution, mouse host; N-cadherin, 1:500 dilution, rabbit host; Actin, 1:10,000, mouse host, BD Transduction Laboratories, San Jose, CA, USA, Catalogue No. 612656). Protein level was measured by electrochemiluminescence (ECL) system (Pierce Biotechnology, Rockford, IL USA).

### Patient datasets and data analysis from REMBRANDT

The past NCI REMBRANDT (used to be at https://caintegrator.nci.nih.gov/rembrandt/login.do, and currently housed in Georgetown University’s G-DOC System ﻿at ﻿ https://gdoc.georgetown.edu/gdoc/) provided de-identified open data on 343 glioma patients through to May 13, 2014. The correlations between E- or N-cadherin expression and OS were checked in samples from the patients as described previously [12]. The construction of graphs was based on the data according to Affymetrix reporters 219,330 at the Highest Geometric Mean Intensity and related survival. Upregulated, downregulated, or intermediate group represented ≥ 2-fold changes in E- or N-cadherin level in comparison with the level of nonglioma samples. Survival differences in groups were estimated by the log-rank test.

### Statistical analyses

All data were analyzed with IBM SPSS Statistics program version 23.0 for Window (Armonk, NY, USA), as previously described [12]. For relationships between E- or N-cadherin expression and WHO tumor grades, chi-square test or Fisher’s exact probability test was used. The effect of single variables on OS or PFS was estimated by univariate and multivariate analyses. Cox’s proportional hazards model was applied to find out the independent prognostic factors. A certain variable that could be influenced by other variables, e.g., postoperative adjuvant therapy, was ruled out from the model. The level of significance was set at *P* < 0.05.

## Results

### Clinical presentation

This series of 92 cases included 42 male patients (45.7%) and 50 female patients (54.3%). The mean age at the time of histological diagnosis was 47.2 years (range: 2 to 84 years). Fifty eight patients (63.0%) underwent gross total resection and 34 patients (37%) underwent subtotal to partial resection. Forty-four patients (47.8%) received concomitant radiation therapy and chemotherapy. The clinicopathological features of our cases are summarized in Table 1.

**Table 1:** Clinicopathologic features of the patients

Variable		Number	Percent
Age (year)	<60	63	68.5%
	≥60	29	31.5%
Sex	Male	42	45.7%
	Female	50	54.3%
WHO grade	I	6	6.5%
	II	28	30.4%
	III	14	15.2%
	IV	44	47.8%
Tumor size	< 4.5 cm	49	53.3%
	≥ 4.5 cm	43	46.7%
Location	Non-eloquent area	45	48.9%
	Near eloquent area	47	51.1%
Edema	None to minimal	42	45.7%
	Moderate to severe	50	54.3%
Cystic change	None	40	43.5%
	Present	52	56.5%
Resection degree	Partial to subtotal	34	37.0%
	Gross total	58	63.0%
Postoperative adjuvant therapy	CT + RT	44	47.8%
	CT alone	1	1.1%
	RT alone	20	21.7%
	None	27	29.3%

### Expression of E- and N-cadherin in gliomas

**Fig. 1:**
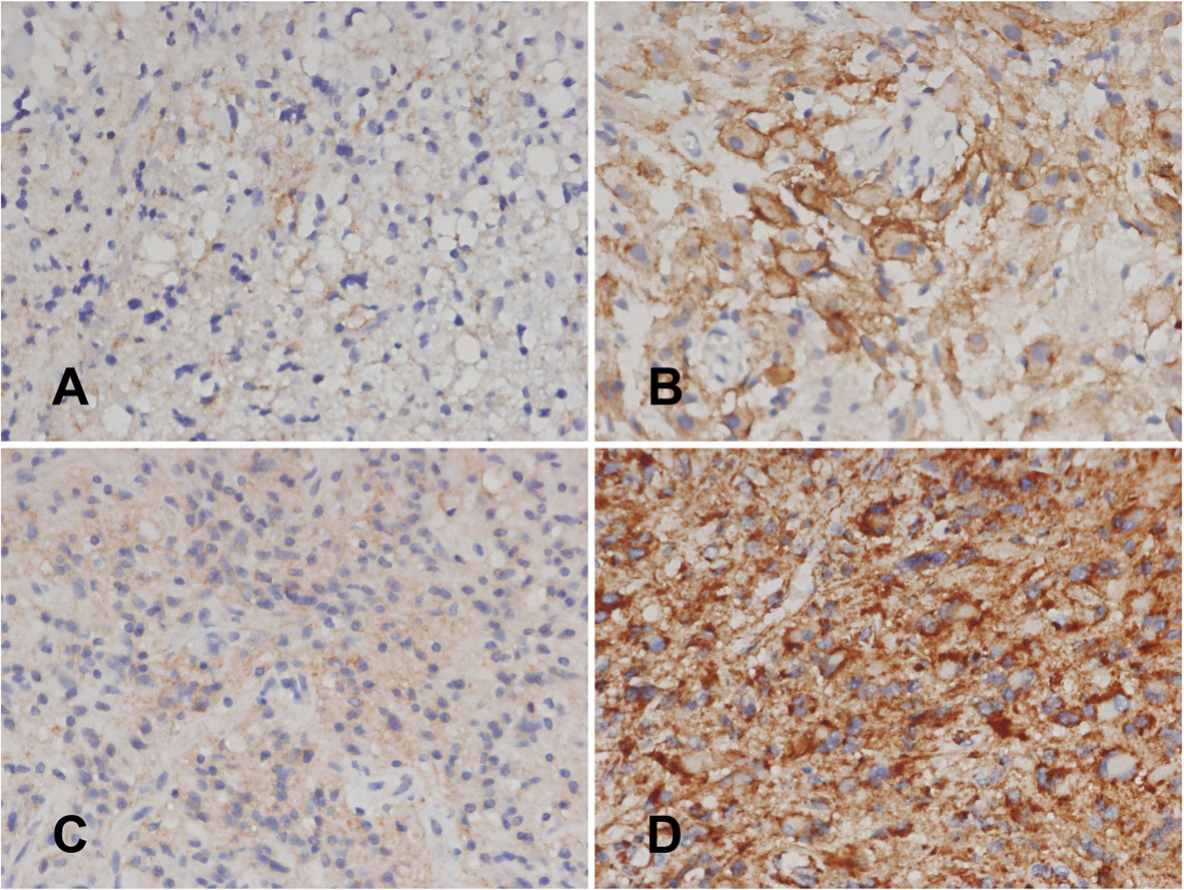
Immunohistochemical findings using E- and N-cadherin antibodies. Most tumor cells showed no to mild expression of E-cadherin (**a**), and only one case displayed strong positivity along the cytoplasmic borders (**b**). Immunohistochemical analysis for N-cadherin showed from mild (**c**) to strong (**d**) positivity in the cytoplasm of the tumor cells

Expression of E- and N-cadherin in gliomas was explored according to WHO tumor grades. E-cadherin expression was observed in eight cases of glioma (Fig. 1a & b). Of 34 low-grade gliomas, three cases showed E-cadherin expression; two cases showed weak expression and one case of subependymal giant cell astrocytoma (SEGA) showed strong expression (Fig. 1b). Five cases out of 58 high-grade gliomas showed E-cadherin immunopositivity, although the staining intensity was weak in all five cases. The correlation between presence of E-cadherin expression and WHO tumor grades (low-grade vs. high-grade) was not significant statistically (*P* = 0.973, Table 2).

**Table 2:** Immunohistochemical expression of E- and N-cadherin in association with WHO tumor grade

Marker	Staining intensity	No. (%)	WHO grade				
			Low (I-II)		High (III-IV)		*P* value
E-cadherin	0	84 (91.3%)	31	31 (36.9%)	53	53 (63.1%)	0.973
	1	7 (7.6%)	2	3 (37.5%)	5	5 (62.5%)	
	2	0 (0.0%)	0		0		
	3	1 (1.1%)	1		0		
N-cadherin	0	11 (12.0%)	5	11 (39.3%)	6	17 (60.7%)	0.759
	1	17 (18.5%)	6		11		
	2	30 (32.6%)	9	23 (35.9%)	21	41 (64.1%)	
	3	34 (37.0%)	14		20		

N-cadherin was expressed in the majority of the glioma cases (cases with staining intensity 1 through 3, 81/92, 88.0%), regardless of the staining intensity (Fig. 1c & d). No expression was found in 6 of 58 high-grade (10.3%) and 5 of 34 low-grade gliomas (14.7%). When the cases were categorized into low-expression group and high-expression group, low expression of N-cadherin was observed in 28 cases (30.5%). N-cadherin was highly expressed in 64 cases (69.5%). Of 58 high-grade tumors, 17 cases displayed low expression of N-cadherin and 41 cases showed high expression. N-cadherin expression was not significantly associated with WHO tumor grades, either (*P* = 0.759).

**Fig. 2:**
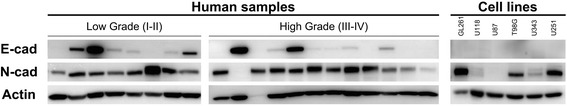
E- and N-cadherin expression in human glioma samples and glioma cell lines. Western blot analysis demonstrated that the majority of human gliomas, both low- and high grades, displayed N-cadherin expression while a small number of gliomas are positive for E-cadherin and that the proportion of positive cases were similar in both groups. Similarly, 5glioma cell lines (U118, U251, T98G, U343, GL261) showed positive bands for N-cadherin

The expressions of E- and N-cadherin were similarly demonstrated by Western blot analysis in the fresh frozen tissues (Fig. 2). Compared to N-cadherin expression, which was detected in the majority of the glioma samples, the number of cases with E-cadherin expression was much smaller. Also, the positive reaction rates of E- and N-cadherin did not differ significantly according to the tumor grades. Five glioma cell lines including U118, T98G, U343, U251 and GL261 showed N-cadherin expression bands by Western blot analysis.

### OS and PFS

**Fig. 3:**
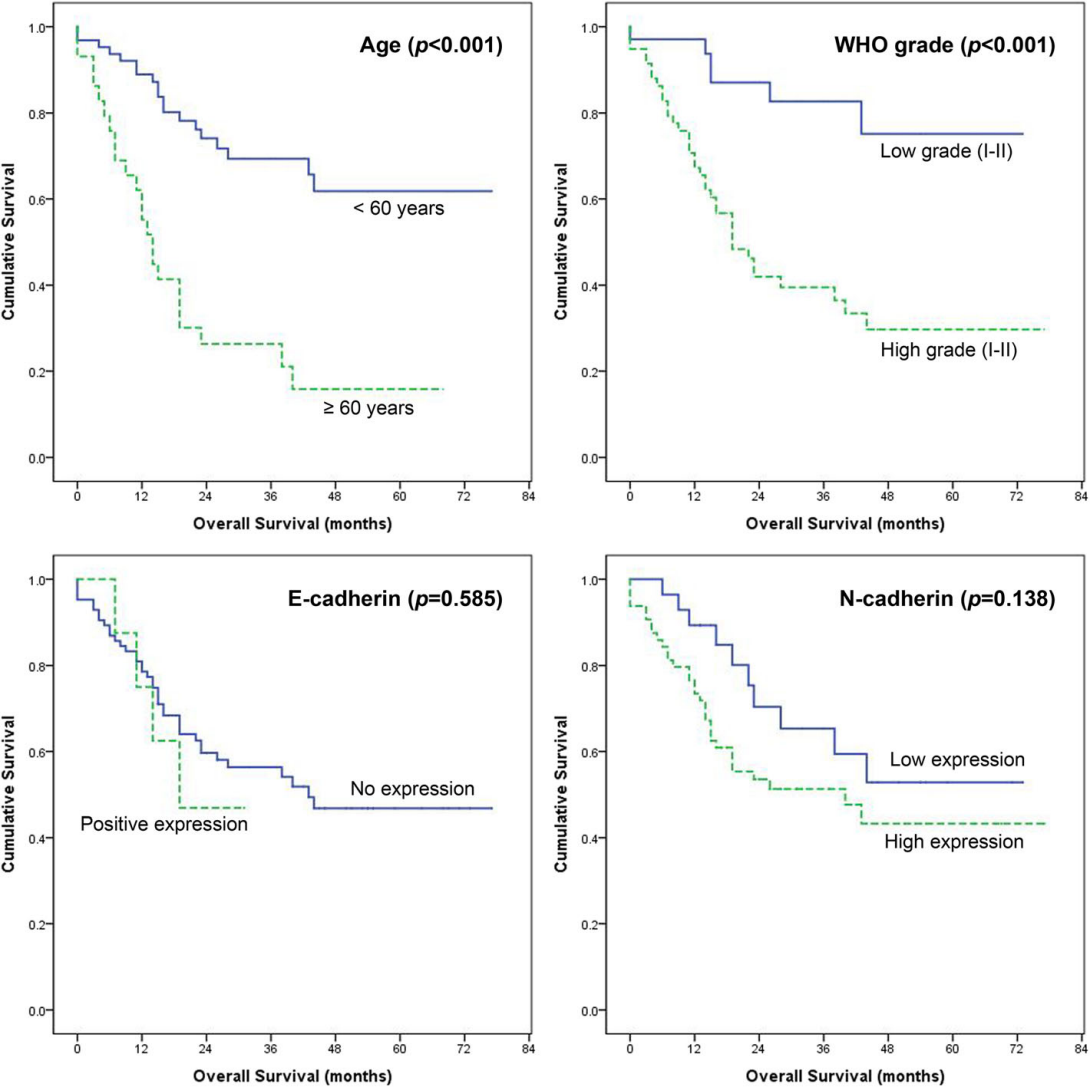
Kaplan–Meier estimates of overall survival according to age, tumor grades and E- and N-cadherin expression level. Age (*P* < 0.001) and WHO tumor grade (*P* < 0.001) showed statistically significance. Expression of E-cadherin and N-cadherin was not related with overall survival of glioma patients

Median OS of all patients was 44.8 months (95% confidence interval (CI): 37.8–51.8 months). The clinical variables of age and WHO tumor grade were significantly associated with longer survival in univariate analysis (both *P* < 0.001) (Fig. 3, Table 3) and multivariate analysis (*P* = 0.002 and *P* = 0.004, respectively). Smaller tumor size was marginally associated with longer survival by univariate analysis (*P* = 0.086) but did not show statistical significance by multivariate analysis (*P* = 0.151). Sex, location, peritumoral edema, cystic or necrotic change, resection degree and E- and N-cadherin expression were not significantly associated with survival benefit. OS by low N-cadherin expression was not statistically significant either by univariate analysis or multivariate analysis (both *P* > 0.05). However, patients with low N-cadherin expression showed longer survival than patients with high expression (49.8 months vs. 42.0 months, Fig. 3 and Table 3).

**Table 3:** Univariate and multivariate analysis for overall survival predictors in patients with glioma

Characteristics		No	Mean (months)	*P*-value (univariate)	*P*-value (multivariate)	Hazard ratio
Age	<60	63	55.4	<0.001	0.002	1
	≥60	29	22.5			2.982
Sex	M	42	43.5	0.962	0.672	1
	F	50	44.7			0.855
WHO grade	Low (I-II)	34	60.7	<0.001	0.004	1
	High (III-IV)	58	34.2			4.016
Tumor size	<4.5	49	50.0	0.086	0.151	1
	≥4.5	43	37.8			1.752
Location	Non-eloquent area	45	31.7	0.141	0.487	1
	Near eloquent area	47	38.4			1.290
Edema	None/minimal	42	35.6	0.581	0.280	1
	Moderate/severe	50	34.4			0.675
Cystic change	None	40	37.5	0.941	0.870	1
	Present	52	34.6			1.062
Resection degree	Partial/subtotal	34	33.6	0.959	0.455	1
	Gross total	58	36.8			1.303
E-cadherin	No	84	45.4	0.585	0.780	1
	Positive	8	21.5			1.181
N-cadherin	Low	28	49.8	0.138	0.456	1
	High	64	42.0			1.362

**Fig. 4:**
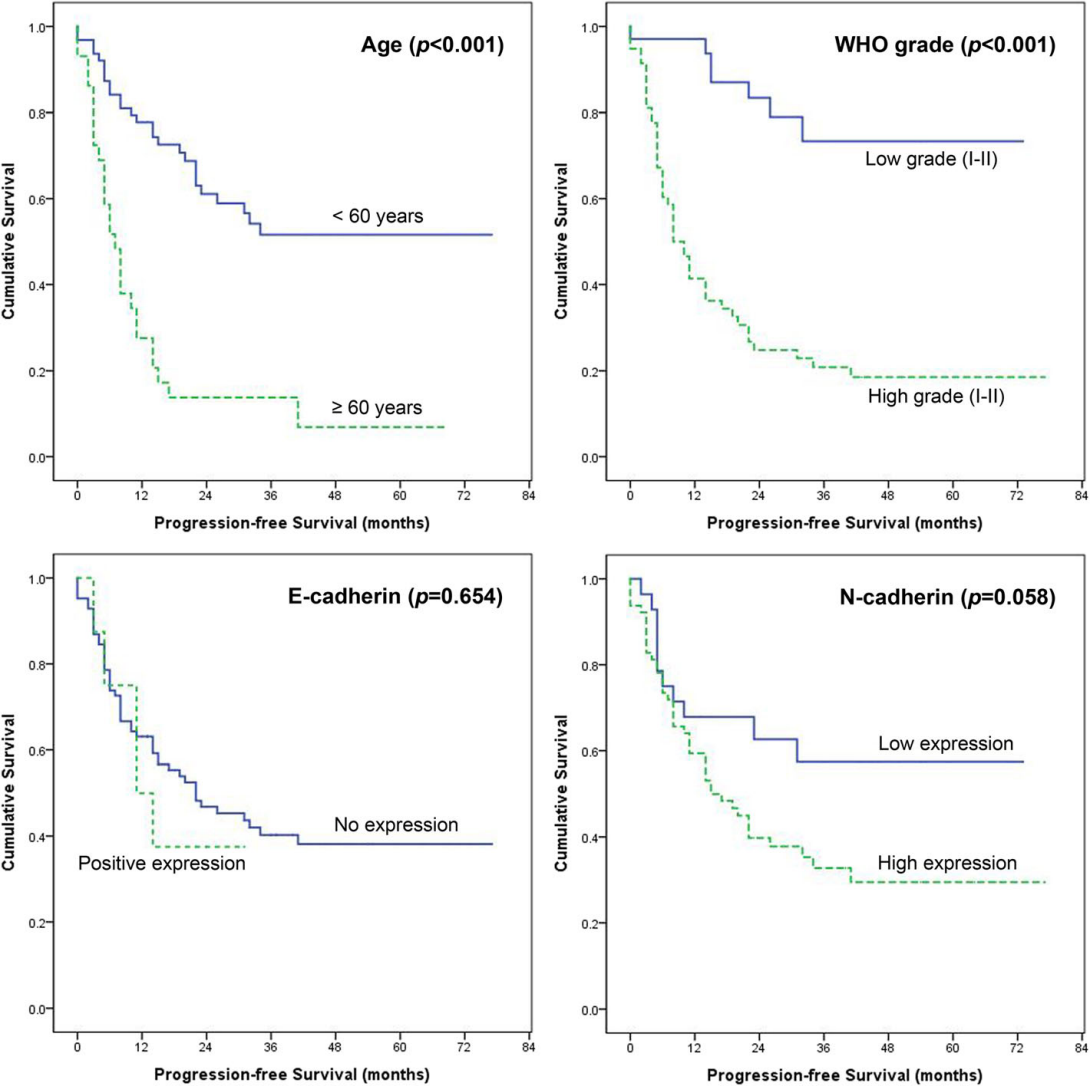
Kaplan–Meier estimates of progression-free survival according to age, tumor grades and E- and N-cadherin expression level. Age (*P* < 0.001) and WHO tumor grade (*P* < 0.001) showed statistically significance. Expression of N-cadherin was related with progression-free survival of glioma patients with marginal statistical significance

PFS was also analyzed in the context of clinical variables. Similarly to the results of OS analysis, only younger age and lower WHO tumor grades showed statistical significance with relevance to the survival benefit by both univariate and multivariate analyses (Fig. 4 and Table 4, all *P* < 0.05). Interestingly, smaller tumor size showed significantly longer PFS (*P* = 0.046) and low N-cadherin expression was marginally associated with survival benefit (*P* = 0.058) by univariate analysis, although both variables did not prove to be independent prognostic factors by multivariate analysis (*P* = 0.255 and *P* = 0.463, respectively). Increased PFS gap between the low N-cadherin expression group and high-expression group compared to OS curves are shown in Fig. 4.

**Table 4:** Univariate and multivariate analysis for progression-free survival predictors in patients with glioma

Characteristics		No	Mean (months)	*P*-value (univariate)	*P*-value (multivariate)	Hazard ratio
Age	<60	63	47.0	<0.001	0.002	1
	≥60	29	13.4			2.673
Sex	M	42	33.1	0.623	0.432	1
	F	50	38.1			0.775
WHO grade	Low (I-II)	34	58.8	<0.001	<0.001	1
	High (III-IV)	58	22.7			5.278
Tumor size	<4.5	49	43.0	0.046	0.255	1
	≥4.5	43	28.4			1.487
Location	Non-eloquent area	45	42.3	0.549	0.913	1
	Near eloquent area	47	44.1			0.966
Edema	None/minimal	42	41.4	0.800	0.794	1
	Moderate/severe	50	45.0			0.920
Cystic change	None	40	45.8	0.840	0.954	1
	Present	52	42.1			1.019
Resection degree	Partial/subtotal	34	42.8	0.746	0.473	1
	Gross total	58	44.4			1.248
E-cadherin	No	28	46.5	0.654	0.838	1
	Positive	64	31.7			1.111
N-cadherin	Low	68	38.2	0.058	0.463	1
	High	24	24.9			1.333

**Fig. 5:**
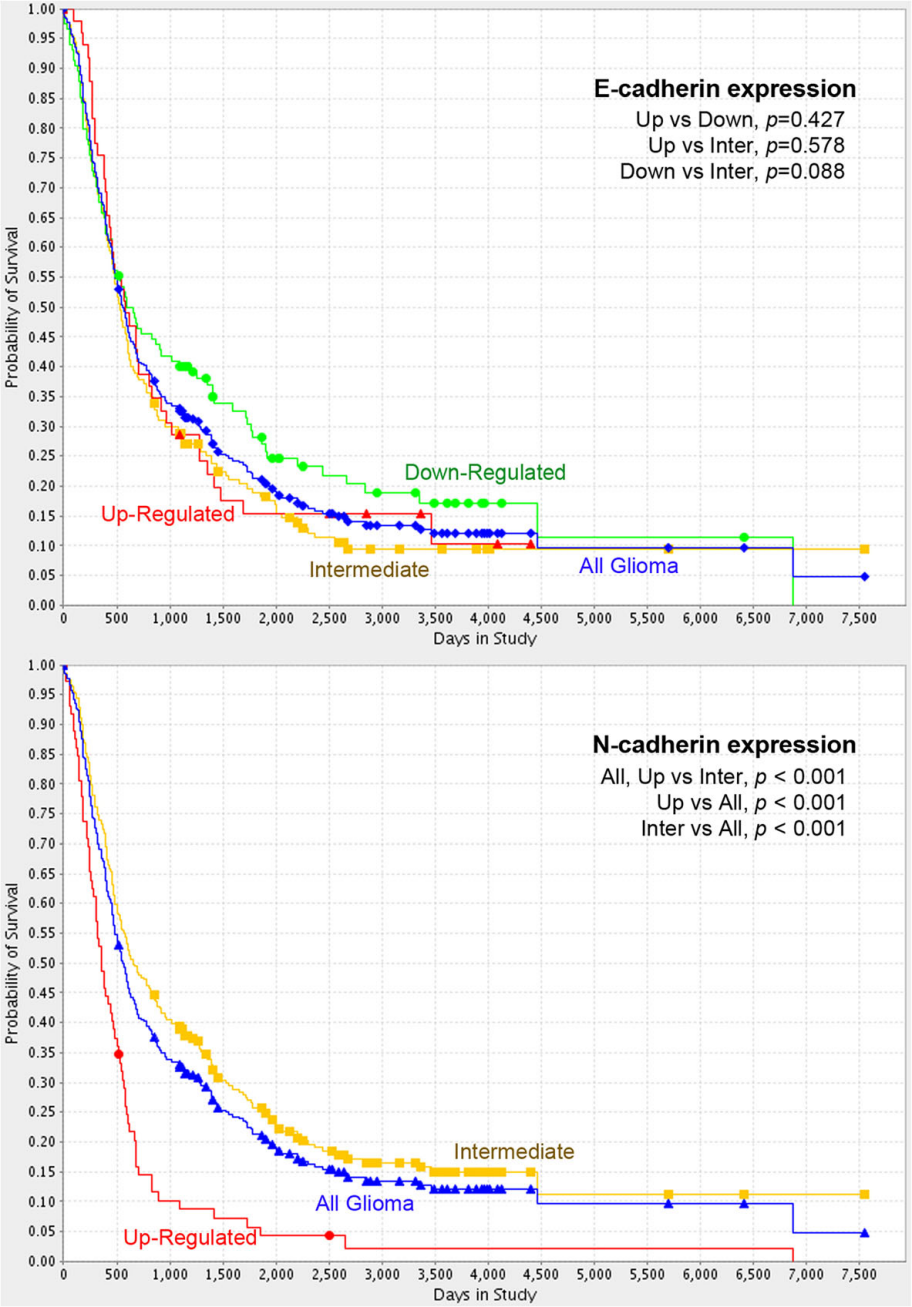
Kaplan–Meier survival analysis according to E-cadherin and N-cadherin expression levels in a cohort of 343 glioma patients in the REMBRANDT database. Overall survival was significantly shorter in patients with high than intermediate N-cadherin expression (*P* < 0.001)

Assessment of 343 glioma patients in the REMBRANDT cohort showed that levels of N-cadherin expression were significantly correlated with OS gaps. Comparison between up-regulated or intermediate N-cadherin expression groups and all glioma group showed statistically significant survival differences (Fig. 5; all *P* < 0.001). With up-regulated N-cadherin expression, OS was significantly shortened. However, significant OS gaps were not related to E-cadherin level, similar to our glioma cohort data.

## Discussion

In the present study, expression of the representative EMT markers, E- and N-cadherin, was investigated in a series of gliomas consisting of WHO grade I through IV tumors to explore the clinical implication with regard to patient survival. Epithelial phenotypes indicated by E-cadherin expression were rarely identified in both low-grade and high-grade tumors, as intuitively expected in non-epithelial malignancies. In comparison, mesenchymal phenotypes denoted by N-cadherin expression were observed in the majority of gliomas through grade I through IV. Although the survival benefit in terms of PFS in the patient group with down-regulated N-cadherin expression showed marginal significance, the survival advantage in the patient group with low N-cadherin expression was increased in the larger REMBRANDT cohort. The current results suggest that the gain of mesenchymal traits in gliomas is boosted by increased N-cadherin expression that is not balanced by E-cadherin alteration.

Glial tumors that lack epithelial phenotypes intrinsically have been observed to rearrange the cytoskeleton, dissimilar to classical EMT of epithelial tumors manifested by E-cadherin to N-cadherin shift [8, 13]. Similar to our data, a prior study reported that the majority of glioblastomas did not show intrinsic E-cadherin expression in a previous study [14]. It has been a very rare occasion to encounter malignant gliomas with E-cadherin expression [17].

Malignant glioma is notable for biological heterogeneity and extreme fatalness, which is fairly connected to its infiltrative attribute. [1, 2]. EMT is a crucial component in early developmental course, tissue repair process and restructuring [18], and takes an important part of tumor advancement [19] and metastasis [4]. Certain transcription factors such as Slug, Snai1, Twist and matrix metalloproteinases, have been reported to be implicated in EMT, and to promote glioma cell migration and invasion [19–22]. Epithelial cell plasticity is rigorously redirected to display increased mesenchymal cadherins including N-cadherin or cadherin-11 and to convert immotile parent epithelial cells to motile cells with enhanced invasive properties [23, 24].

In cancerous transformations, the acquisition of EMT traits is closely linked to the proceeding of dedifferentiation and gain of stem cell status [4]. As supported by important experimental findings in epithelial malignancies including colon, pancreatic and breast cancer, induction of EMT can co-induce stem cell properties, thereby connecting cell motility and stem cell-like programs [4, 25, 26]. Malignant gliomas turned out to have cancer stem cell population recently [27]. However, the co-existence of stem cell and EMT features during the progression of glioblastoma has been described lately [9]. Aside from metastasis, which is rare in gliomas, dedifferentiated phenotype in the residual tumor cell population at the invasive front is coupled with malignant transformation in the recurrent tumor. Despite removal of extensive tumor volume based on grossly detectable levels, microscopic foci of remnant tumor cells beyond the resection margins bring about eventual tumor recurrence, not infrequently accompanied by advance into a higher-grade glioma. With the most infiltrative phenotype of the cells in the invasion front far off the resection margins, the tumor cells are prone to proliferate and to progress after a variable dormant period by means of stem cell characteristics in the remaining population. A previous study reported the enhanced expression of stem cell factors in paired primary and recurrent glioma samples by unsupervised clustering analysis of gene expression profiling [28]. The concurrence of a stem cell status with EMT features in glioblastoma, either via the β–catenin pathway or KITENIN mediation, has been recently described [12, 13].

Increased mesenchymal traits are considered to be a pivotal molecular event that leads to enhanced malignancy in gliomas [8]. Based on genome-scale analysis of large cohorts of glioblastomas, four different subgroups were identified that are dependent on neural differentiation [10, 11]. Glioblastomas categorized into the mesenchymal subtype displayed by far shorter overall and progression-free survival periods, related with extensive aggressiveness indicated by multifocality or therapeutic resistance against radio- and chemo-therapy [11]. Accordingly, the clinical consequence of invasive phenotype induced by EMT is evident. A previous study proposed that a small population of glioma cells undergo molecular events that bring about cytoskeletal reorganization and apoptotic resistance. As a result, the tumor cells become highly motile and invasive and then evolve into the treatment-resistant condition [8]. Compared to metastases of most carcinomas in which the eventual process recapitulate the organization of the primary tumors [29], the evolutional changes in glioma progression do not involve the organization represented by restoration of E-cadherin expression. Instead, glioma progression seems to incorporate strengthened mesenchymal phenotypes in relevance to up-regulated N-cadherin expression and resultant therapeutic resistance. In addition, glioblastoma clusters other than the mesenchymal subtype acquire mesenchymal traits over recurrent episodes [11]. An evolutional change with increased mesenchymal phenotype appears to be a frequent episode with regard to disease progression, alike cancer cells using EMT mechanism during the advance to a more aggressive status [8, 30]. However, further investigation needs be performed to reveal the relation between N-cadherin or other EMT markers/inducers and key parameters for new 2016 WHO glioma classification, such as IDH1 mutation, 1p/19q co-deletion or MGMT promoter methylation [31].

## Conclusions

The present study shows that E- and N-cadherin expressions in glioma have limited value as a survival predictor. However, N-cadherin expression showed prognostic implication with marginal significance in our glioma cohort and by far more significant prognostic meaning in the larger REMBRANDT data. The EMT process accompanied by enhanced N-cadherin expression may contribute the biological aggressiveness in glioma by increased mesenchymal phenotype that is supported by variably unfavorable prognostic outcome.

## Abbreviations

EMT: Epithelial-mesenchymal transition
IHC: Immunohistochemistry
MRI: Magnetic resonance imaging
OS: Overall survival
PFS: Progression-free survival

## References

[1] Louis DN, Ohgaki H, Wiestler OD, Cavenee WK, Burger PC, Jouvet A, Scheithauer BW, Kleihues P (2007). The 2007 WHO classification of tumours of the central nervous system. Acta Neuropathol.

[2] Rousseau A, Mokhtari K, Duyckaerts C (2008). The 2007 WHO classification of tumors of the central nervous system - what has changed?. Curr Opin Neurol.

[3] Kleihues P, Louis DN, Wiestler OD, Burger PC, Scheithauer BW, Louis DN, Ohgaki H, Wiestler OD, Cavenee WK (2007). WHO grading of tumours of the central nervous system. WHO classification of tumours of the central nervous system.

[4] Brabletz T (2012). To differentiate or not--routes towards metastasis. Nat Rev Cancer.

[5] Nagaishi M, Paulus W, Brokinkel B, Vital A, Tanaka Y, Nakazato Y, Giangaspero F, Ohgaki H (2012). Transcriptional factors for epithelial-mesenchymal transition are associated with mesenchymal differentiation in gliosarcoma. Brain Pathol.

[6] Guo Y, Zi X, Koontz Z, Kim A, Xie J, Gorlick R, Holcombe RF, Hoang BH (2007). Blocking Wnt/LRP5 signaling by a soluble receptor modulates the epithelial to mesenchymal transition and suppresses met and metalloproteinases in osteosarcoma Saos-2 cells. J Orthop Res.

[7] Cosset E, Hamdan G, Jeanpierre S, Voeltzel T, Sagorny K, Hayette S, Mahon FX, Dumontet C, Puisieux A, Nicolini FE, Maguer-Satta V (2011). Deregulation of TWIST-1 in the CD34+ compartment represents a novel prognostic factor in chronic myeloid leukemia. Blood.

[8] Kahlert UD, Nikkhah G, Maciaczyk J (2013). Epithelial-to-mesenchymal(−like) transition as a relevant molecular event in malignant gliomas. Cancer Lett.

[9] Kalluri R, Weinberg RA (2009). The basics of epithelial-mesenchymal transition. J Clin Invest.

[10] Phillips HS, Kharbanda S, Chen R, Forrest WF, Soriano RH, Wu TD, Misra A, Nigro JM, Colman H, Soroceanu L, Williams PM, Modrusan Z, Feuerstein BG, Aldape K (2006). Molecular subclasses of high-grade glioma predict prognosis, delineate a pattern of disease progression, and resemble stages in neurogenesis. Cancer Cell.

[11] Verhaak RG, Hoadley KA, Purdom E, Wang V, Qi Y, Wilkerson MD, Miller CR, Ding L, Golub T, Mesirov JP, Alexe G, Lawrence M, O'Kelly M, Tamayo P, Weir BA, Gabriel S, Winckler W, Gupta S, Jakkula L, Feiler HS, Hodgson JG, James CD, Sarkaria JN, Brennan C, Kahn A, Spellman PT, Wilson RK, Speed TP, Gray JW, Meyerson M, Getz G, Perou CM, Hayes DN, Cancer Genome Atlas Research Network (2010). Integrated genomic analysis identifies clinically relevant subtypes of glioblastoma characterized by abnormalities in PDGFRA, IDH1, EGFR, and NF1. Cancer Cell.

[12] Lee KH, Ahn EJ, Oh SJ, Kim O, Joo YE, Bae JA, Yoon S, Ryu HH, Jung S, Kim KK, Lee JH, Moon KS (2015). KITENIN promotes glioma invasiveness and progression, associated with the induction of EMT and stemness markers. Oncotarget.

[13] Kahlert UD, Maciaczyk D, Doostkam S, Orr BA, Simons B, Bogiel T, Reithmeier T, Prinz M, Schubert J, Niedermann G, Brabletz T, Eberhart CG, Nikkhah G, Maciaczyk J (2012). Activation of canonical WNT/beta-catenin signaling enhances in vitro motility of glioblastoma cells by activation of ZEB1 and other activators of epithelial-to-mesenchymal transition. Cancer Lett.

[14] Mikheeva SA, Mikheev AM, Petit A, Beyer R, Oxford RG, Khorasani L, Maxwell JP, Glackin CA, Wakimoto H, Gonzalez-Herrero I, Sánchez-García I, Silber JR, Horner PJ, Rostomily RC (2010). TWIST1 promotes invasion through mesenchymal change in human glioblastoma. Mol Cancer.

[15] Qi S, Song Y, Peng Y, Wang H, Long H, Yu X, Li Z, Fang L, Wu A, Luo W, Zhen Y, Zhou Y, Chen Y, Mai C, Liu Z, Fang W (2012). ZEB2 mediates multiple pathways regulating cell proliferation, migration, invasion, and apoptosis in glioma. PLoS One.

[16] Yang HW, Menon LG, Black PM, Carroll RS, Johnson MD (2010). SNAI2/slug promotes growth and invasion in human gliomas. BMC Cancer.

[17] Lewis-Tuffin LJ, Rodriguez F, Giannini C, Scheithauer B, Necela BM, Sarkaria JN, Anastasiadis PZ (2010). Misregulated E-cadherin expression associated with an aggressive brain tumor phenotype. PLoS One.

[18] Kalluri R, Neilson EG (2003). Epithelial-mesenchymal transition and its implications for fibrosis. J Clin Invest.

[19] Thiery JP (2002). Epithelial-mesenchymal transitions in tumour progression. Nat Rev Cancer.

[20] Kang Y, Massague J (2004). Epithelial-mesenchymal transitions: twist in development and metastasis. Cell.

[21] Qiao B, Johnson NW, Gao J (2010). Epithelial-mesenchymal transition in oral squamous cell carcinoma triggered by transforming growth factor-beta1 is snail family-dependent and correlates with matrix metalloproteinase-2 and -9 expressions. Int J Oncol.

[22] Radisky ES, Radisky DC (2010). Matrix metalloproteinase-induced epithelial-mesenchymal transition in breast cancer. J Mammary Gland Biol Neoplasia.

[23] Tran NL, Nagle RB, Cress AE, Heimark RL (1999). N-Cadherin expression in human prostate carcinoma cell lines. An epithelial-mesenchymal transformation mediating adhesion withStromal cells. Am J Pathol.

[24] Zeisberg M, Neilson EG (2009). Biomarkers for epithelial-mesenchymal transitions. J Clin Invest.

[25] Morel AP, Lievre M, Thomas C, Hinkal G, Ansieau S, Puisieux A (2008). Generation of breast cancer stem cells through epithelial-mesenchymal transition. PLoS One.

[26] Mani SA, Guo W, Liao MJ, Eaton EN, Ayyanan A, Zhou AY, Brooks M, Reinhard F, Zhang CC, Shipitsin M, Campbell LL, Polyak K, Brisken C, Yang J, Weinberg RA (2008). The epithelial-mesenchymal transition generates cells with properties of stem cells. Cell.

[27] Singh SK, Hawkins C, Clarke ID, Squire JA, Bayani J, Hide T, Henkelman RM, Cusimano MD, Dirks PB (2004). Identification of human brain tumour initiating cells. Nature.

[28] Kwon SM, Kang SH, Park CK, Jung S, Park ES, Lee JS, Kim SH, Woo HG (2015). Recurrent Glioblastomas reveal molecular subtypes associated with mechanistic implications of drug-resistance. PLoS One.

[29] Brabletz T, Jung A, Spaderna S, Hlubek F, Kirchner T (2005). Opinion: migrating cancer stem cells - an integrated concept of malignant tumour progression. Nat Rev Cancer.

[30] Iwadate Y (2016). Epithelial-mesenchymal transition in glioblastoma progression. Oncol Lett.

[31] Louis DN, Perry A, Reifenberger G, von Deimling A, Figarella-Branger D, Cavenee WK, Ohgaki H, Wiestler OD, Kleihues P, Ellison DW (2016). The 2016 World Health Organization classification of tumors of the central nervous system: a summary. Acta Neuropathol.

